# A Prospective Study on Functional Outcomes of Serial Cast Correction in Congenital Talipes Equinovarus (CTEV) by Ponseti Method

**DOI:** 10.7759/cureus.35987

**Published:** 2023-03-10

**Authors:** Ramavtar Saini, Anshu Sharma, Divyaraj Ravalji, Kuldeep Baisoya, Garima Sharma

**Affiliations:** 1 Orthopaedics, Geetanjali Medical College and Hospital, Udaipur, IND; 2 Obstetrics and Gynaecology, Geetanjali Medical College and Hospital, Udaipur, IND

**Keywords:** congenital talipes equinovarus, percutaneous tendoachilles tenotomy, ponseti technique, pirani score, idiopathic clubfoot, congenital talipes equinovarus (ctev)

## Abstract

Introduction: Congenital talipes equinovarus (CTEV), often known as clubfoot, is one of the most common congenital deformities of the foot and ankle, affecting one in every 1,000 live births. The deformity has four components: ankle equinus, hindfoot varus, forefoot adductus, and midfoot cavus. The Ponseti casting technique of CTEV management has proven to be more effective, generating higher positive outcomes and lesser complications than conventional surgical methods.

Methods: This study was conducted in a tertiary care teaching hospital centre in southern Rajasthan, India. Twenty cases with 31 feet of untreated clubfoot were included. The deformity was scored according to Pirani's scoring system. Manipulation and serial corrective casts were applied at weekly intervals according to Ponseti's method. Percutaneous tendoachilles tenotomy was done whenever required (guided by Pirani score). Final results were calculated using the Pirani score, noted before putting the patient on foot abduction orthosis.

Results: In our study, the average number of casts required for full correction was 6.5. Heel cord tenotomy was required in 27 feet (87%) to achieve full correction. Final Pirani score significantly improved from an average of 4.8 on presentation to 0.055 after completion of casting.

Conclusion: We conclude that the Ponseti technique of CTEV correction significantly reduces the need for invasive surgical procedures along with being exceedingly safe, effective, and affordable. The Ponseti technique of cast correction is crucial and provides a painless, plantigrade, cosmetically acceptable foot with higher functional outcomes and minimal complications.

## Introduction

Congenital talipes equinovarus (CTEV), also known as clubfoot, is one of the most common complex congenital deformities of the foot and ankle in infants. The deformity has four components: ankle equinus, hindfoot varus, forefoot adductus, and midfoot cavus [1]. CTEV occurs in one in every 1,000 live births, predominantly in males (male-to-female ratio of 2:1), and approximately half of them have bilateral clubfoot [2]. Children who are born in underdeveloped nations with clubfoot deformity may receive inadequate treatment when compared to the developed nations, which lowers their quality of life.

The controversies around CTEV management have been in existence since the beginning. Hippocrates, in around 400 BC, provided the earliest descriptions of clubfoot care and was the first to recommend bandaging and moderate massage as a treatment modality for clubfoot [3].

Over the past century, two major concepts have guided the development of clubfoot treatment. The first is the widespread adoption of the concepts of manipulation, strapping, and serial plaster treatment, while the second is in favour of a variety of surgical techniques for clubfoot correction. However, in recent practices, the Ponseti method could lead to a functioning, painless, and morphologically normal foot. Due to several drawbacks of surgical release procedures of CTEV, such as stiffness, recurrence, over-correction, discomfort, and ankylosis, there appears to be a resurgence of interest in conservative techniques for the management of congenital clubfoot [4,5].

The conservative treatment of CTEV was developed by Dr. Ignacio Ponseti, and it begins within a few weeks after birth. It consists of serial corrective manipulation with the aid of using constrained operative intervention (percutaneous Achilles tenotomy). It is based on the pathoanatomy and basic kinematics of the deformity and effectively realigns clubfoot in infants without invasive surgeries [1]. It has become especially vital in developing countries, where the operative centres are out of reach and fewer in number with the added disadvantage of being cost-ineffective.

The first treatment of CTEV should always be non-surgical, starting as early as possible after birth. The treatment mainly focuses on correcting all of the components of clubfoot to reap painless, plantigrade, pliable, and cosmetically and functionally ideal feet within a short period of time and taking into consideration the low socio-economic backgrounds of the parents and children [5]. If there is no improvement, then most surgeons prefer a soft tissue release procedure. The major disadvantages of the soft tissue release procedure include stiffness and its cost ineffectiveness [6,7].

The aim of this study was to analyse the functional outcomes of serial cast correction in CTEV by the Ponseti method. The primary objective was to study the effectiveness of Ponseti's technique of plaster cast application in the management of idiopathic clubfoot. The secondary objective was to assess the severity of deformity using the Pirani scoring system.

## Materials and methods

This prospective interventional study was conducted at a tertiary care medical teaching institute in southern Rajasthan, India. Ethical clearance was obtained from the Institutional Ethics Committee, Geetanjali Medical College and Hospital, Udaipur, Rajasthan (GU/HREC/EC/2021/1933). Infants from birth to 12 months of age with idiopathic clubfoot deformity were included in the study after obtaining informed written consent from their parents. Children above the age of 12 months or with a previous history of clubfoot surgery and atypical or secondary clubfoot were excluded from the study. Based on the inclusion and exclusion criteria, 20 babies with 31 feet of untreated idiopathic clubfoot were selected from the CTEV clinic.

A thorough general and local examination was carried out and a detailed history was obtained. The deformity was scored according to Pirani's scoring system [8]. Manipulation and serial corrective casts were applied at weekly intervals according to Ponseti's technique. The sequence of deformity correction was cavus followed by adduction and varus deformity. While the equinus deformity was corrected after the midfoot deformity corrections as guided by Pirani's score and usually requires percutaneous heel cord tenotomy. While applying the cast, a pacifier was used to calm the child. Cases were regularly followed up at weekly intervals and serial corrective casting was done for six to eight weeks as per requirement. Replacement of plaster casts at shorter intervals gives better results. On each visit, before applying the cast, improvement in deformity was documented using Pirani’s scoring system. Under general anaesthesia, percutaneous tendoachilles tenotomy was performed whenever required (guided by Pirani’s score) and the final cast was applied for three weeks. Denis Browne splints were applied for three months. Modified CTEV shoes were given to children who had started weight-bearing on lower limbs. Instructions regarding night-time bracing were given for three to four years. Parental education and constant reassurance to accept long-term brace treatment helped in maintaining the correction, thereby preventing relapses.

Pirani scoring system

In 1995, Pirani described a system for grading clubfoot [8]. It is composed of six different physical examination findings, three at the midfoot level and three at the hindfoot, each scored 0 for no abnormality, 0.5 for moderate abnormality, and 1.0 for severe abnormality. Each foot was assigned a total score ranging from zero to six; a higher score indicating a more severe deformity means a severely abnormal foot. For grading the extent of involvement, three components are included in the midfoot score: curved lateral border, medial crease, and coverage of the talar head. Three components included in the hindfoot score are the posterior crease, rigid equinus, and empty heel (Table 1).

**Table 1:** Pirani scoring system

Parameters	Mild	Moderate	Severe
Midfoot scores			
Curved lateral border	0	0.5	1
Deep medial crease	0	0.5	1
Uncovering of talar head	0	0.5	1
Hindfoot scores			
Empty heel	0	0.5	1
Posterior crease	0	0.5	1
Rigid equinus	0	0.5	1

The aim of Ponseti's technique [1,9,10] is to achieve biomechanical realignment of the foot. Correction of CTEV using the Ponseti technique can be divided into two phases; first, the treatment phase, during which the deformities are corrected, and second, the maintenance phase, during which a brace is used to prevent the recurrence of deformity.

The treatment phase starts as early as possible after birth, and till that time, regular corrective manipulation of the foot after each feed is carried out by the mother or grandmother. Throughout the duration of the appointment, the infant is kept comfortable.

First cast

**Figure 1:**
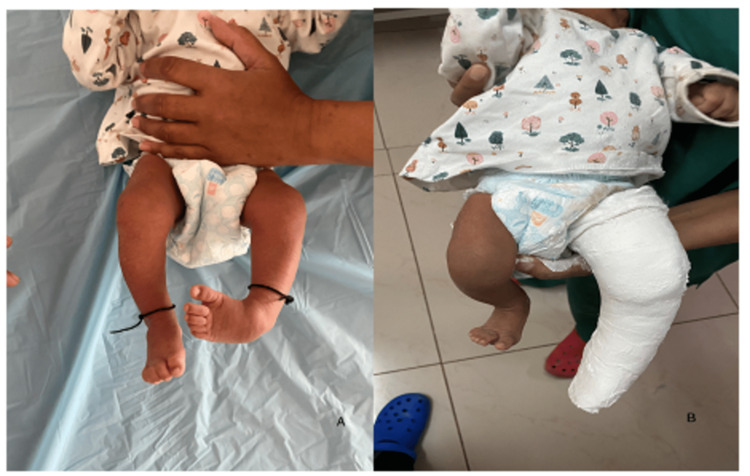
Ponseti technique of CTEV correction: the first cast for cavus correction The first cast is aimed at the alignment of the forefoot and midfoot by supinating the forefoot and some gentle abduction. Figure A is showing clubfoot with cavus deformity. Figure B is showing above knee cast in the corrected position. CTEV: congenital talipes equinovarus.

The treatment phase starts with the first cast aiming to correct the cavus deformity (high medial arch). The cause of cavus deformity is the pronated forefoot in relation to the hindfoot. Cavus is often flexible in neonates, thus the only correction needed to produce a proper longitudinal arch of the foot is to supinate the forefoot by raising the first metatarsal and abduct the foot gently while stabilizing the talus by placing the thumb over the lateral part of its head. It is followed by the application of a well-padded above-knee plaster cast by holding the position and moulding it well. The parents are advised to return after a week and complications associated with the cast are explained thoroughly (Figure 1).

Subsequent casts

After the removal of the first cast, the severity of the cavus deformity is assessed and if the cavus is not corrected, the first step is repeated. If the cavus has been corrected, then after a short period of manipulation, the next plaster cast is applied. These casts are aimed at the gradual correction of abduction, heel varus, and ankle equinus deformity. The talus is stabilized by placing the thumb over the lateral part of its head and holding the supinated foot in abduction while applying the well-padded above-knee plaster cast. At each weekly follow-up, the previous cast is removed, the foot is evaluated for deformity correction using the Pirani score, manipulated, and the next plaster cast is applied.

**Figure 2:**
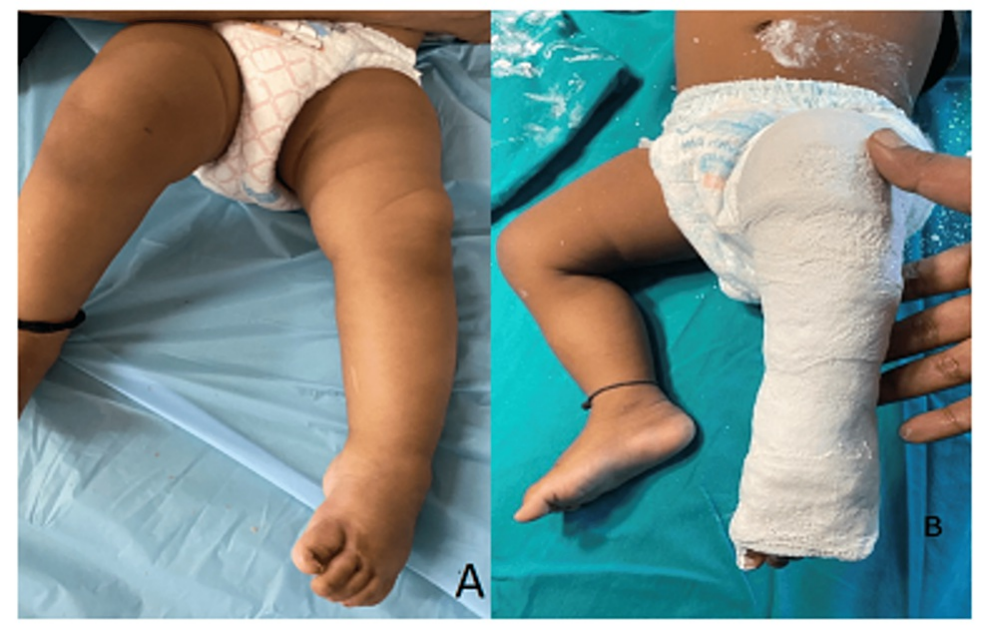
Ponseti technique of CTEV correction: subsequent casts Subsequent casts are aimed at the correction of midfoot inversion and heel varus. Figure A is showing corrected cavus deformity. Figure B is showing above knee cast in the corrected position. CTEV: congenital talipes equinovarus.

An important point in the Ponseti technique is that the heel is never directly manipulated. The correction of the heel varus and ankle equinus takes place simultaneously because of the coupling of the tarsal bones. After the fifth or sixth cast, a complete correction could be achieved (Figure 2).

Achilles tendon tenotomy

**Figure 3:**
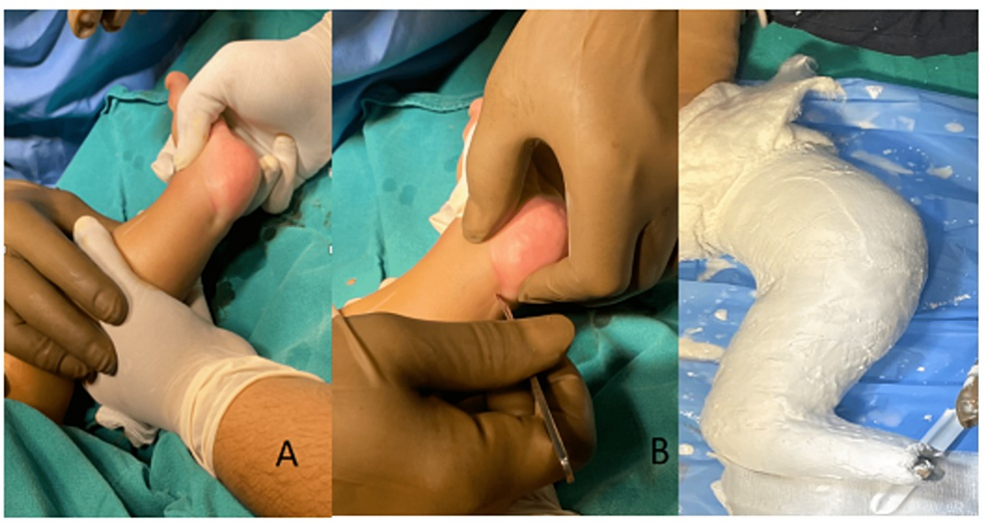
Ponseti technique of CTEV correction: Achilles tendon tenotomy Role of Achilles tendon tenotomy in the correction of residual equinus deformity. Figure A is showing residual equinus at the ankle joint. Figure B is showing the procedure of tenotomy. Figure C is showing post-tenotomy cast in dorsiflexion at the ankle joint. CTEV: congenital talipes equinovarus.

In the majority of children, there is some equinus deformity at the ankle, which persists. Correction of this residual deformity is achieved with a percutaneous surgical release of the Achilles tendon, which allows dorsiflexion at the ankle joint. The timing of the tenotomy is guided by the Pirani score. Tenotomy was performed when the hind-foot score was more than 1 and the mid-foot score was less than 1. After the tenotomy, the final cast is applied with the foot in 70 degrees of abduction and 10-15 degrees of dorsiflexion. This cast is removed after three weeks and the final Pirani score is calculated (Figure 3).

Brace

**Figure 4:**
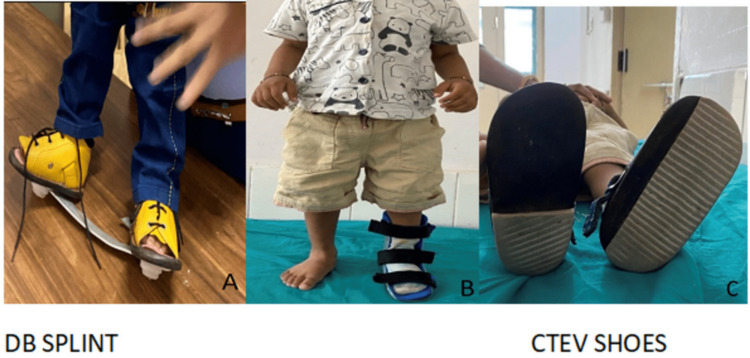
Ponseti technique of CTEV correction: post-correction orthosis to maintain the correction Post-correction orthosis. Figure A is showing Denis Browne (DB) splint. Orthopaedic CTEV shoes front view (B) and bottom view (C). CTEV: congenital talipes equinovarus.

After the removal of the final cast, a brace is used to maintain the foot in its corrected position. The brace is a bar attached to a straight open-toed shoe. For the first three months following the removal of the final cast, the brace should be worn for 23 hours a day. After this, the child should wear the brace for a total of 14 to 16 hours per day, including 12 hours at night and two to four hours during the day time. This protocol is followed until the child is three to four years old. If the bracing protocol is not correctly followed, the relapse rate may exceed more than 80% (Figure 4).

**Figure 5:**
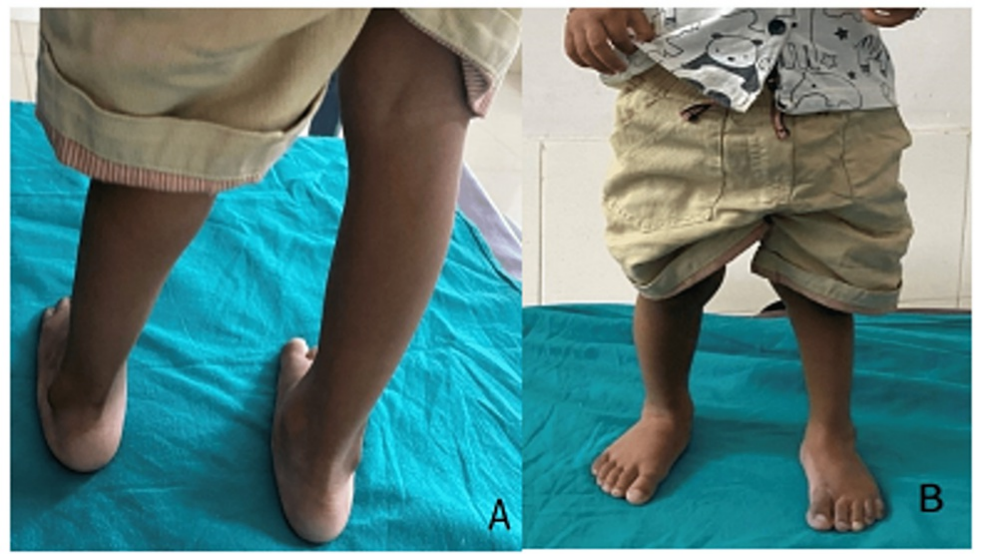
Ponseti technique of CTEV correction: Clinical images at final follow-up Clinical images of both lower limbs following successful correction of CTEV deformity (left foot). Figure A is the view from the back side and figure B is the front view. CTEV: congenital talipes equinovarus.

Clinical images of our case at the final follow-up after the end of the treatment phase with the continuation of the maintenance phase (Figure 5).

## Results

A total of 20 cases with 31 idiopathic clubfeet were included in our study after obtaining institutional ethical committee clearance. Out of 20 cases, 15 patients were male with a male-to-female ratio of 3:1. Predominantly bilateral involvement was seen in 55% of cases, while 20% of cases were on the left side and 25% were on the right side. A total of 50% of cases were presented within the first two months of age. Fourteen cases (70%) were of firstborn children and six cases (30%) were of birth order second or above. Out of 20 cases, only one case (5%) was born from consanguineous marriage and 95% were born from a non-consanguineous marriage. All the cases were freshly diagnosed idiopathic clubfeet and had no previous history of any conservative or operative treatment (Table 2).

**Table 2:** Demographic variables of cases

Variables		Number of cases (%)
Sex	Male	15 (75%)
	Female	05 (25%)
Side of involvement (out of 31 feet)	Bilateral	11 (55%)
	Left	04 (20%)
	Right	05 (25%)
Age at presentation (in months)	0-2 months	10 (50%)
	>2-6 months	06 (30%)
	>6 months	04 (20%)
Birth order	Firstborn	14 (70%)
	Second or above	06 (30%)
Consanguinity	Consanguineous marriage	01 (05%)
	Non-consanguineous marriage	19 (95%)

The mean initial Pirani score was 4.8 with a maximum value of six and the minimum recorded score was 2.5. In the present study, the correction was achieved by casting only in four cases (12.90%) and 27 cases (87.10%) required casting with heel cord tenotomy. The numbers of casts required for correction in 16 cases (51.62%) were five to six while in 15 cases (48.38%), seven to eight casts were required for correction; the average number of casts for full correction was 6.5. In the present study, one case (3.23%) developed superficial blister formation due to casting while in 31 cases (96.67%), no complications were reported (Table 3).

**Table 3:** Treatment and complication-related variables

Variables		Number of cases (%)
Treatment modality	Casting only	04 (12.90%)
	Casting and heel cord tenotomy	27 (87.10%)
Number of casts required for full correction	5-6 casts	16 (51.62%)
	7-8 casts	15 (48.38%)
Complications	Superficial blister formation	01 (3.23%)
	No complication	30 (96.77%)

In the present study, the final mean Pirani score recorded was 0.055, with the p-value (p < 0.001) being highly significant and indicating the effectiveness of the Ponseti method in the treatment of CTEV (Table 4).

**Table 4:** Relationship between Pirani score at initial presentation and after final cast removal A p-value of less than 0.001 is highly significant and indicates the effectiveness of the Ponseti method in the treatment of congenital talipes equinovarus.

	Pirani score (before treatment)	Pirani score (after treatment)
Min-Max	2.00-6.00	0.00-1.00
Mean ± SD	5.016 ± 0.96	0.103 ± 0.241
P-value	P < 0.001 (Pirani score significantly decreased)	

## Discussion

Clubfoot is a complex musculoskeletal deformity of the foot requiring scrupulous and consistent efforts of the surgeon as well as parents for its correction. Of patients receiving scheduled serial cast repair, 85-90% have reported having satisfactory functional results. In our study, all 20 cases were classified using the Pirani scoring system, which helped us in the assessment of the severity of the deformity. Other classification systems like Carroll or Dimeglio are there but the Pirani scoring system provides some light on the prognosis of CTEV and helps in its management.

Out of the 20 patients, 10 (50%) infants presented within the first two months of life, six cases (30%) were presented between the third to sixth months, and the remaining four patients (20%) were presented between the seventh month and one year of age. This may suggest a deficient referral system and a lack of awareness among parents and guardians.

In the present study, there is male preponderance. Fifteen out of 20 infants were male with a male-to-female ratio of 3:1. This is similar to the series of Yamamoto [11] and Chesney et al. [12], reflecting a higher number of cases in males. This can be due to the existing prejudices, biases, and social norms that favour the male gender in our region.

In this study, bilateral involvement was seen in 55% of cases. A total of 25% of cases were on the left side and 20% were on the right side. In the study conducted by Guruprasath et al. [13], bilaterality was observed in 57.89% of cases while 26.3% were right-sided and 15.78% were left-sided.

In the present study, 14 cases (70%) were of firstborn children, which is in accordance with various other studies conducted by Pulak et al. [14] and Yamamoto [11]. This signifies that congenital clubfoot was more common in firstborn children. In the present study, all 20 cases had no associated conditions. In contrast, Guruprasath et al. [13] observed that 10.52% of cases had associated conditions that included cleft lip (5.26%), developmental dysplasia of the hip (2.63%), and omphalocele (2.63%).

In the present study, the numbers of casts required for correction in 16 feet (52%) were five to six and 15 feet (48%) required seven to eight casts for correction, and the average number of casts for full correction was 6.5. We observed that presentation during the second half of the infancy period and higher initial Pirani score required more casts. The average number of casts required in the study by Laaveg et al. [15] was seven. Replacement of plaster casts at shorter intervals and fewer casts per foot give better results, and therefore, it has been adopted by a number of orthopaedics.

In our study, the maximum initial Pirani score recorded was 6, the minimum score recorded was 2.5, and 4.8 was the mean initial Pirani score. These findings are comparable with Syed et al. [16] and Pulak et al. [14].

Heel cord tenotomy is done for equinus deformity only after correction of forefoot adduction and heel varus. Of the patients in our study, 87% required tenotomy (27 feet); 90% of the patients in the study conducted by Pirani et al. [17] underwent tenotomy while 91% of patients required tenotomy in Dobbs et al.'s [3] series. In our study, we observed that tenotomy was usually required in patients who had a Pirani score of 5 or more at presentation.

In the present study, one case (3.13%) out of 31 feet experienced superficial blister formation, which recovered after the application of adequate soft padding while further casting and allowing the skin to heal. In the study conducted by Lehman [18], 10.2% of cases reported complications. Guruprasath et al. [13] reported a complication rate of 13.15%, which included superficial sores and crowding of toes. These were managed by soft padding and allowing enough space for the toes, mainly the dorsum for free toe movements.

In the present study, the final mean Pirani score recorded was 0.055, with the p-value (p < 0.001) being highly significant and indicating the effectiveness of the Ponseti method in the treatment of CTEV. In a study by Thacker et al. [19], the final Pirani score was 0.00 at the final follow-up, indicating successful correction of the clubfoot deformity by the Ponseti technique.

## Conclusions

We conclude that the Ponseti technique significantly reduces the need for invasive surgical procedures along with being exceedingly safe, effective, and affordable. Due to its high efficiency and low cost, the Ponseti technique of cast correction is crucial and avoids surgical complications, and provides patients with a painless, plantigrade, cosmetically acceptable foot with higher functional outcomes. Constant reassurance and motivation to the parents to accept long-term brace treatment help in maintaining the correction, thereby preventing relapses. According to our study, the Ponseti method of serial cast correction is an ideal method for the correction of idiopathic CTEV deformity.
